# Vitamin B1 Helps to Limit *Mycobacterium tuberculosis* Growth *via* Regulating Innate Immunity in a Peroxisome Proliferator-Activated Receptor-γ-Dependent Manner

**DOI:** 10.3389/fimmu.2018.01778

**Published:** 2018-08-16

**Authors:** Shengfeng Hu, Wenting He, Xialin Du, Yulan Huang, Yuling Fu, Yalong Yang, Chuxuan Hu, Silin Li, Qinshu Wang, Qian Wen, Xinying Zhou, Chaoying Zhou, Xiao-Ping Zhong, Li Ma

**Affiliations:** ^1^School of Laboratory Medicine and Biotechnology, Institute of Molecular Immunology, Southern Medical University, Guangzhou, China; ^2^Division of Allergy and Immunology, Department of Pediatrics, Duke University Medical Center, Durham, NC, United States

**Keywords:** *Mycobacterium tuberculosis*, vitamin B1, macrophages, peroxisome proliferator-activated receptor-γ, adjuvant

## Abstract

It is known that vitamin B1 (VB1) has a protective effect against oxidative retinal damage induced by anti-tuberculosis drugs. However, it remains unclear whether VB1 regulates immune responses during *Mycobacterium tuberculosis* (MTB) infection. We report here that VB1 promotes the protective immune response to limit the survival of MTB within macrophages and *in vivo* through regulation of peroxisome proliferator-activated receptor γ (PPAR-γ). VB1 promotes macrophage polarization into classically activated phenotypes with strong microbicidal activity and enhanced tumor necrosis factor-α and interleukin-6 expression at least in part by promoting nuclear factor-κB signaling. In addition, VB1 increases mitochondrial respiration and lipid metabolism and PPAR-γ integrates the metabolic and inflammatory signals regulated by VB1. Using both PPAR-γ agonists and deficient mice, we demonstrate that VB1 enhances anti-MTB activities in macrophages and *in vivo* by down-regulating PPAR-γ activity. Our data demonstrate important functions of VB1 in regulating innate immune responses against MTB and reveal novel mechanisms by which VB1 exerts its function in macrophages.

## Introduction

Tuberculosis, caused by the bacterium *Mycobacterium tuberculosis* (MTB), remains a major global health challenge and is the leading cause of mortality among infectious diseases worldwide. MTB is estimated to infect one-third of the world’s population, but only 10% of infected individuals show symptoms and develop clinical disease (1). Upon infection with MTB, several factors contribute to the disease outcome, with cell-mediated immunity representing one of the most critical determinants (2). As the first line of immune defense against MTB, macrophages provide a major habitat for MTB to remain dormant in the host for several years. Triggered upon MTB infection, multiple inflammatory signaling pathways in macrophages are activated to initiate a tailored immune response toward the invading pathogen (3). Depending on phenotype and function, macrophages can polarize to several macrophage subsets, such as classically activated macrophages (M1 macrophages), alternatively activated macrophages (M2 macrophages), regulatory macrophage, tumor-associated macrophages, and myeloid-derived suppressor cells (4). The M1 phenotype displays an inflammatory profile such as expressing CD86 and MHC-II, secreting pro-inflammatory cytokines tumor necrosis factor α (TNFα) and interleukin-6 (IL-6), and producing iNOS-dependent reactive nitrogen intermediates (4). M2 macrophages can be further subdivided into three subgroups: M2a, M2b, and M2c (5) and are marked by expressing CD206 and arginase 1 (6). The productions of pro-inflammatory cytokines TNF-α and IL-6 play a crucial role in pathogen clearance (7).

However, active tuberculosis occurs when the infection is no longer contained by the immune system. Tuberculosis can lead to weight loss and micronutrient deficiencies by increasing nutritional requirements, changing metabolic processes, decreasing appetite, and reducing food intake (8). Poor nutritional status is more common in people with active tuberculosis than in people without tuberculosis. Likewise, tuberculosis is more common in individuals with poor nutritional status (9). Nutrient deficiencies can result in immunosuppression and dysregulation of immune responses. Particularly, deficiencies in certain nutrients can impair phagocytic function in innate immunity including cytokine production, as well as cell-mediated immunities (10, 11), which can increases the susceptibility to active tuberculosis and delays recovery (12).

Vitamins are organic compounds and essential nutrients required by an organism in limited amounts. An increasing number of studies have begun to explore the mechanisms by which vitamins regulate immunity and their effects as adjuvant to treat tuberculosis (13, 14). Vitamin (V) A, D, and E are the most widely studied, and the mechanisms by which they regulate immunity have been partly elucidated (15, 16). Vitamin B1 (VB1) (also known as thiamin or thiamine) is needed for the metabolism of carbohydrates, but cannot be produced in humans, and thus it is an essential nutrient. It is known that vitamin B1 had a protective effect against oxidative retinal damage induced by antituberculotics (17). However, it is unclear whether VB1 participates in the immune regulation process during MTB infection.

Peroxisome proliferator-activated receptor γ (PPAR-γ), a member of the lipid-activated nuclear receptor family, has been implicated in the differentiation, and lipid metabolism of innate immune cells including macrophages and involved in inflammatory responses (18). In macrophages, PPARs integrate metabolic and inflammatory signaling to PPAR-γ function as an important “molecular switch” in regulating immune responses and nutrient metabolism during MTB infection (18–20).

In this study, we demonstrated that VB1 promoted the protective immune response in mice to enhance their resistance to MTB infection *via* regulating macrophage function. VB1 promoted the polarization of macrophages into strongly microbicidal, classically activated phenotype, and increased their expression of TNF-α and IL-6 *via* regulating NF-κB signaling in a PPAR-γ-dependent manner during MTB infection.

## Materials and Methods

### Animals

Specific pathogen-free C57BL/6J mice, 6 weeks old, were purchased from the Experimental Animal Center of Southern Medical University. PPAR-γ^floxp/floxp^ (PPAR-γ^fl/fl^) and Lyz2-cre mice were purchased by Shanghai Research Center for Model Organisms (Shanghai, China). All animal experiments in this study were carried out in accordance with the recommendations in the Guide for the Care and Use of Laboratory Animals of the National Institutes of Health. All experimental protocols were reviewed and approved by the Medical Ethics Board and the Biosafety Management Committee of Southern Medical University (approval number L2015123).

### Infection of Mice and Colony-Forming Units (CFUs)

6-week-old female and male mice were exposed to 1 × 10^7^ CFUs of MTB H37Rv (ATCC 27294, the same below) in an Inhalation Exposure System (Glas-Col, USA), which delivers ~200 bacteria to the lung per animal. At 24 h after infection, bacterial titers in the lungs of at least two mice were determined to confirm the dose of MTB H37Rv inoculation. After infection, VB1 (Sigma-Aldrich, USA), isoniazid (INH), or water were orally administered daily until the indicated times. The VB1 solution (7.3 g/L) was prepared by dissolving 220 mg VB1 in 30 mL water. The INH solution (2.0 g/L) and water serve as positive and negative controls, respectively. Oral administration was started from 1 day after infection and performed every day until the specified time. For VB1-treated mice, the VB1 dose (200 μL/mouse) was equivalent to 20 µg VB1/100 g body weight. INH or water was given in the same manner as that for VB1-treated mice. Rosiglitazone (Sigma-Aldrich, USA) was administered intraperitoneally in 125 µL of corn oil at 20 mg/kg. Sham mice received corn oil only. Bacterial burden was determined by plating serial dilutions of spleen and lung homogenates onto 7H10 agar plates (BD Biosciences, USA) with 10% OADC. Plates were incubated in 5% CO_2_ at 37°C for 3~4 weeks before counting colonies.

### Lung Cells Isolation

Lung cell suspensions were prepared by perfusing cold saline containing heparin through the heart, removed, and sectioned in ice-cold medium. Dissected lung tissue was incubated in 0.7 mg/mL collagenase IV and 30 µg/mL DNase [Sangon Biotech (Shanghai), China] at 37°C for 30 min. Digested lungs were disrupted by passage through a 70-µm nylon cell strainer, treated with red blood cell lysis buffer, and processed over a 40:80% Percoll (GE Healthcare) gradient. The resulting cell suspension was washed and counted.

### Culture of Bone Marrow-Derived Macrophages (BMDMs), Mycobacterial Infection, and Stimulation of VB1

Bone marrow cells were taken from C57BL/6J mice and placed on cell culture dishes (96 mm × 22 mm; CELLTER, China) at 37°C/5% CO_2_ in DMEM (Corning, USA) containing 10% fetal bovine serum (FBS; Corning, USA). The cells differentiated into macrophages induced by granulocyte macrophage colony-stimulating factor (100 ng/mL; PeproTech, USA) until the seventh day. BMDMs were placed on a 12-well cell culture plates (CELLTER) for 48 h at 37°C/5% CO_2_ in DMEM containing 10% FBS. Then cells were persistently infected with MTB H37Rv until the indicated time. VB1 (20 µM) was added every 24 h.

### Fluorescence-Activated Cell Sorting (FACS) Analysis

For surface staining, BMDMs or lungs cells were harvested, washed, and stained for 30 min on ice with mixtures of fluorescently conjugated mAbs or isotype-matched controls. mAbs of mice were as follows: FITC-anti-F4/80, APC-anti-CD80, PE-Cy7-anti-CD86, PE-anti-MHC-II, Percp-Cy5.5-anti-CD11b, Pacific Blue-anti-Gr-1 (eBioscience, USA). Cell phenotype was analyzed by flow cytometry on a flow cytometer (BD LSR II) (BD Biosciences, USA). Data were acquired as the fraction of labeled cells within a live-cell gate and analyzed using FlowJo software (Tree Star). All gates were set on the basis of isotype-matched control antibodies.

### Enzyme-Linked Immunosorbent Assay (ELISA)

Lungs were homogenized in 2 mL PBS + 0.05% Tween 80. Homogenized tissue supernatants were filtered (0.22 μm). Cell culture supernatants were collected and assayed for cytokines. Cytokine production was measured by ELISA of mouse TNF-α and IL-6 (ExCell Bio, China) according to the manufacturer’s protocol.

### Real-Time PCR

Total RNA was isolated using TRIzol reagent (Thermo Fisher Scientific) according to the manufacturer’s recommendations. For mRNA, first-strand cDNA synthesis was performed using RevertAid First-Strand cDNA Synthesis Kit (Thermo Fisher Scientific). An Eppendorf Master Cycle Realplex2 and SYBR Green PCR Master Mix (Applied Biosystems, USA) were used for real-time PCR (40 cycles). PCR products were then separated by electrophoresis through a 1% agarose gel and were visualized by being stained with ethidium bromide. The forward primer and reverse primer for mTNF-α were 5′-CACAGAAAGCATGATCCGCGAC-3′ and 5′-TGCCACAAGCAGGAATGAGAAGAG-3′. The forward primer and reverse primer for mIL-6 were 5′-GTCCGGAGAGGAGACTTCAC-3′ and 5′-CTGCAAGTGCATCATCGTTGT-3′. The forward primer and reverse primer for mβ-Actin were 5′-GATTACTGCTCTGGCTCCTAGC-3′ and 5′-GACTCATCGTACTCCTGCTTGC-3′.

### Western Blotting

Cells were washed three times with ice-cold PBS and then lysed in lysis buffer containing 1 mM phenylmethylsulfonyl fluoride, 1% (vol/vol) protease inhibitor cocktail (Sigma-Aldrich, USA), and 1 mM DTT. Equal amounts (20 mg) of cell lysates were resolved using 8–15% polyacrylamide gels transferred to PVDF membrane. Membranes were blocked in 5% non-fat dry milk in PBST and incubated overnight with the respective primary antibodies at 4°C. These respective primary antibodies list are as follows: Phospho-NF-κB p65 (Ser536) (Clone: 93H1; CST, USA), NF-κB p65 (Clone: D14E12; CST), Phospho-Akt (Ser473) (Clone: D9E; CST), Akt (Clone: C67E7; CST), Phospho-p38 MAPK (Thr180/Tyr182) (Clone: D3F9; CST), p38 MAPK (Clone: D13E1; CST), Phospho-p44/42 MAPK (Erk1/2) (Thr202/Tyr204) (Clone: D13.14.4E; CST), p44/42 MAPK (Erk1/2) (Clone: 137F5; CST), Phospho-JNK (Thr183/Tyr185) (Clone: G9; CST), SAPK/JNK (CST), GAPDH (Clone: D16H11; CST), PPARγ (Clone:81B8; CST), and SUMO-1 (Clone: C9H1, CST). The membranes were incubated at room temperature for 1 h with appropriate HRP-conjugated secondary antibodies and visualized with Plus-ECL (PerkinElmer, CA, USA) according to the manufacturer’s protocol.

### Oxygen Consumption Rate (OCR) Analysis

Mitochondrial OCR in intact cells was measured using the XF-24 analyzer (Seahorse Bioscience, USA) as described in the manufacturer’s instructions. Briefly, BMDMs were seeded into XF-24 microplates and then maintained at 37°C in a non-CO_2_ incubator for at least 1 h before assay. ATP turnover and maximal uncoupled OCRs were determined by treating the cells with oligomycin (1 mmol/L) (Sigma-Aldrich) or carbonyl cyanide 4-(trifluoromethoxy) phenylhydrazone (FCCP; 1 mmol/L) (Sigma-Aldrich), respectively. Rotenone and antimycin A (1 mmol/L each) (Sigma-Aldrich) were used to inhibit complex 1- and complex 3-dependent respiration. OCR was normalized to protein content. Each experimental condition was analyzed using four to six biological replicates.

### Lipid Body Staining and Enumeration

Cells and infection were performed as above. The cells were fixed with 4% paraformaldehyde for 5 min, stained with 0.5% Oil red O for 30 min at room temperature, and counterstained with hematoxylin to stain nuclei. Cells were rinsed with PBS, mounted on glass slides, and imaged. Lipid bodies ere enumerated by light microscopy with a 3,100 objective lens for 50 consecutive macrophages on each slide.

### Co-Immunoprecipitations

Bone marrow-derived macrophages were seeded on 100-mm dishes at 1 × 10^6^ cells per dish. Cells were treated with VB1 for 24 h. Cells were lysed in 1% digitonin (Calbiochem) buffer (20 mM Tris–HCl, 150 mM NaCl, 1% digitonin) containing protease inhibitors (Roche). Cleared supernatants were incubated with 10 mg of anti-PPAR-γ antibody, followed by incubation with immobilized protein G (Pierce). The beads were washed four times by 1% digitonin lysis buffer and immunoprecipitates were eluted with SDS sample buffer by boiling for 5 min.

### *In Vitro* MTB Killing Assay

Bone marrow-derived macrophages were allowed to adhere to 12-well flat bottom plates at 5 × 10^5^ cells per well and infected with MTB H37Rv at an MOI of 5 for 1 h at 37°C with 5% CO_2_, then wells were extensively washed with pre-warmed PBS to remove non-adherent bacteria. The cells were incubated at 37°C with 5% CO_2_ for indicated time, and then were lysed in 1 mL of distilled water. Bacterial burden was determined by plating serial dilutions onto 7H10 agar plates supplemented with 10% OADC. Plates were incubated at 37°C in 5% CO_2_ for 3 weeks before counting colonies. All infections were performed in triplicate.

### Statistics

All experiments were performed at least twice. When shown, multiple samples represent biological (not technical) replicates of mice randomly sorted into each experimental group. No blinding was performed during animal experiments. Determination of statistical differences was performed with Prism 5 (Graphpad Software, Inc.) using unpaired two-tailed *t*-tests (to compare two groups with similar variances), or one-way ANOVA with Bonferonni’s multiple comparison test (to compare more than two groups).

## Results

### VB1 Led to Decreased Mycobacterial Growth in Mice *via* Regulating Function of Macrophages

**Figure 1:**
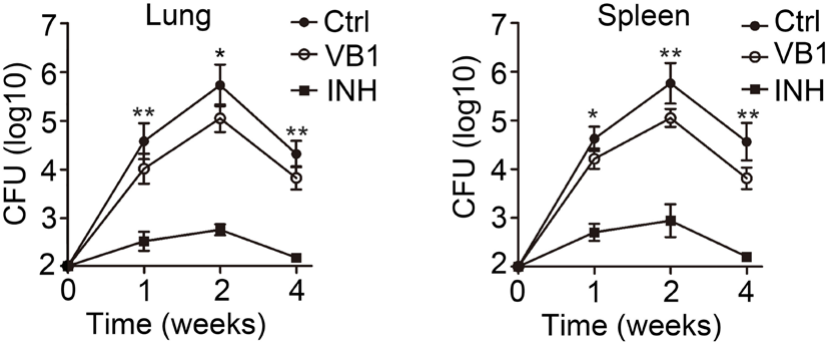
The anti-bacillus effect of vitamin B1 (VB1) in mice with *Mycobacterium tuberculosis* infection. C57BL/6J mice were infected with H37Rv (~200 bacteria/mouse). Oral administration with water (Ctrl), VB1, and INH (*n* = 15 mice/group) was started from the day after infection (day 1) and continued for 1, 2, and 4 weeks alternatively. The lungs and spleens were analyzed at indicated time. Colony-forming units (CFUs) were obtained from the lung and spleen cell lysates by serial dilution and plating on 7H10 agars in triplicate. The colonies were counted after 4 weeks. Data shown are the mean ± SD. *P < 0.05 and **P < 0.01. Data are representative of three independent experiments with similar results.

Because the role of VB1 in antibacterial growth was unclear, we first investigated whether VB1 might affect MTB infection *in vivo*. We infected wild-type mice with MTB H37Rv and orally administrated either VB1, isoniazid (INH), or water (control group, Ctrl) into the infected mice for 1, 2, and 4 weeks, followed by measurement of MTB burden in the lungs and spleens of infected mice. As expected, INH treatment greatly reduced MTB CFU in mice at all the times examined. Importantly, MTB CFUs were constantly lower in VB1-treated mice than control mice at all indicated time points (Figure 1), suggesting that VB1 treatment enhanced containment of MTB growth was significantly suppressed by VB1 treatment.

**Figure 2:**
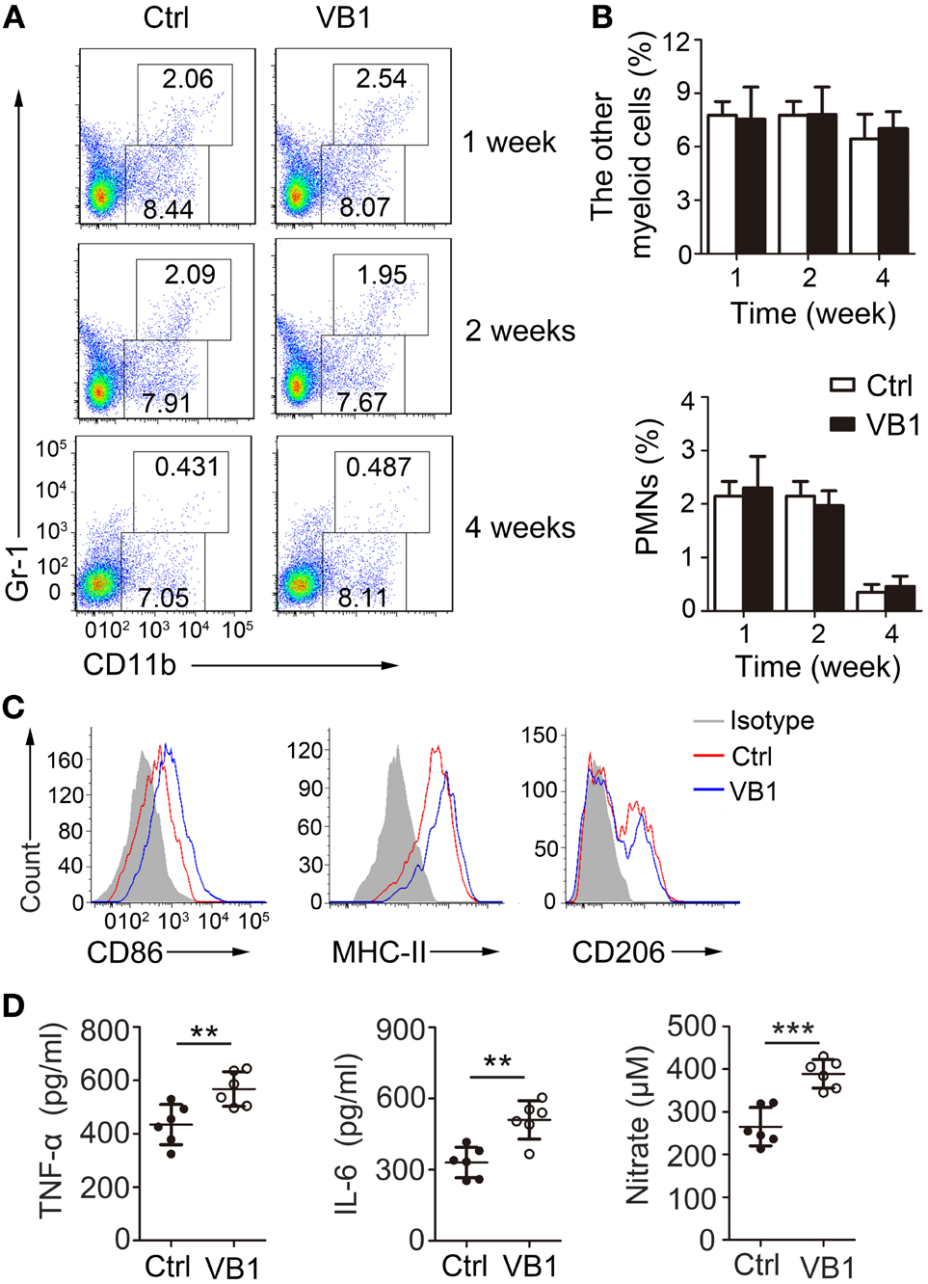
The pro-inflammatory effect of vitamin B1 (VB1) in lungs of *Mycobacterium tuberculosis* (MTB)-infected mice. Lung cells from H37Rv-infected mice treated with VB1 or untreated were harvested at 1 week, 2 weeks, and 4 weeks after infection. **(A)** The percentage of myeloid cells is displayed as dot plots. **(B)** The percentages of other myeloid cells and polymorphonuclear cells in lungs were shown. **(C)** The expressions of CD86, MHC-II, and CD206 were detected *via* flow cytometry at 4 weeks. **(D)** Concentration of tumor necrosis factor α (TNF-α) and interleukin-6 (IL-6) in lungs (homogenized in 2 mL PBS and 0.05% Tween 80) from mice with MTB infection at 1 weeks were detected by enzyme-linked immunosorbent assay. Data shown are the mean ± SD. **P < 0.01 and ***P < 0.001. Data are representative of three independent experiments with similar results.

We next wanted to explore how VB1 promoted an effective immune response against MTB infection. Because VB1 treatment reduced MTB burden at 1 week after infection when adaptive immunity has not been activated during MTB infection (21), we hypothesized that VB1 might affect innate immune immunity against MTB. Since macrophages and neutrophils are the major types of immune cells that kill and eliminate MTB at an early stage of infection, we determined the percentage of immune cells in the lungs of MTB-infected mice treated with VB1 or water 7 days after infection by FACS analysis. We found that VB1 had no effect on the percentages and numbers of CD11b^+^Gr1^−^ monocyte–macrophages and CD11b^+^Gr1^+^ polymorphonuclear cells in lungs from MTB-infected mice (Figures 2A,B; Figure S1A in Supplementary Material). However, we found that macrophages showed increased expression levels of CD86 and MHC-II, which are characteristic features of classically activated macrophages, but decreased expression levels of CD206, which is the characteristic feature of alternatively activated macrophages, from VB1-treated mice infected with MTB H37Rv compared to those obtained from control mice (Figure 2C; Figure S1B in Supplementary Material). The increased levels of TNF-α, IL-6, and nitrate were detected in lung homogenates of VB1-treated mice (Figure 2D). Together these observations indicate that VB1 could regulate the functions of macrophages to promote the protective immune response of the mice during MTB infection.

### VB1 Promoted the Innate Immune Response of Macrophages After Mycobacterial Infection *In Vitro*

**Figure 3:**
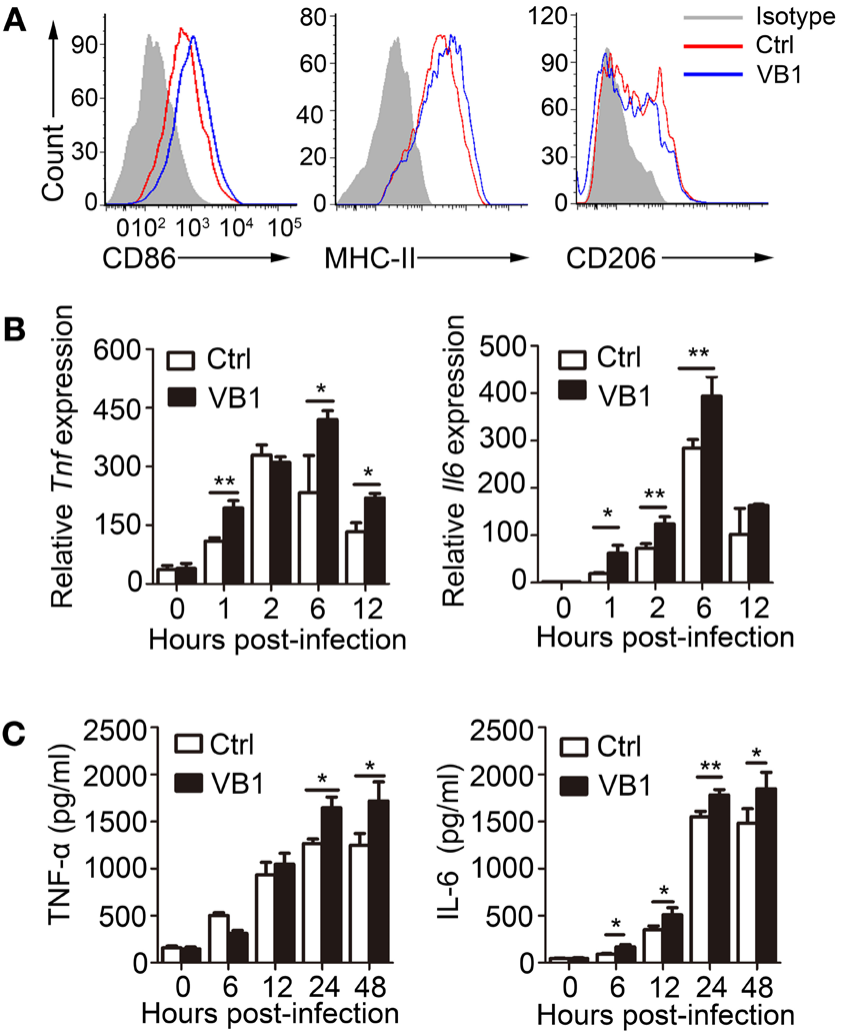
The pro-inflammatory effect of vitamin B1 (VB1) on bone marrow-derived macrophages (BMDMs) after *Mycobacterium tuberculosis* (MTB) infection. BMDMs were pretreated with VB1 or phosphate buffer saline (Ctrl) for 24 h followed by MTB H37Rv infection (MOI 5). **(A)** Expressions of CD86, MHC-II, and CD206 were detected *via* flow cytometry after infection at 24 h. **(B)** Tumor necrosis factor α (TNF-α) and interleukin-6 (IL-6) mRNA expression determined by real-time PCR. **(C)** TNF-α and IL-6 secretion for indicated time points was measured by enzyme-linked immunosorbent assay. Data shown are the mean ± SD. *P < 0.05, **P < 0.01. Data are representative of three independent experiments with similar results.

To determine if VB1 affected the function of macrophages directly during MTB infection, we pretreated BMDMs with VB1 *in vitro* for 24 h, followed by MTB H37Rv infection. VB1-treated BMDMs displayed enhanced upregulation of CD86 and MHC-II expression (Figure 3A; Figure S2A in Supplementary Material), increased TNF-α and IL-6 expression at both mRNA (Figure 3B) and protein (Figure 3C) levels, and increased nitrate production (Figure S2B in Supplementary Material) compared with PBS-treated group, indicating enhanced M1 polarization. By contrast, CD206 expression and the activity of arginase-I were decreased in VB1-treated BMDMs at 24 h after MTB infection (Figure 3A; Figure S2B in Supplementary Material), suggesting reduced M2 polarization. Overall, these results demonstrate that VB1 promotes classically activated polarization and pro-inflammatory cytokine production of macrophages after mycobacterial infection *in vitro*.

### VB1 Regulates the NF-κB Signal After Mycobacterial Infection

**Figure 4:**
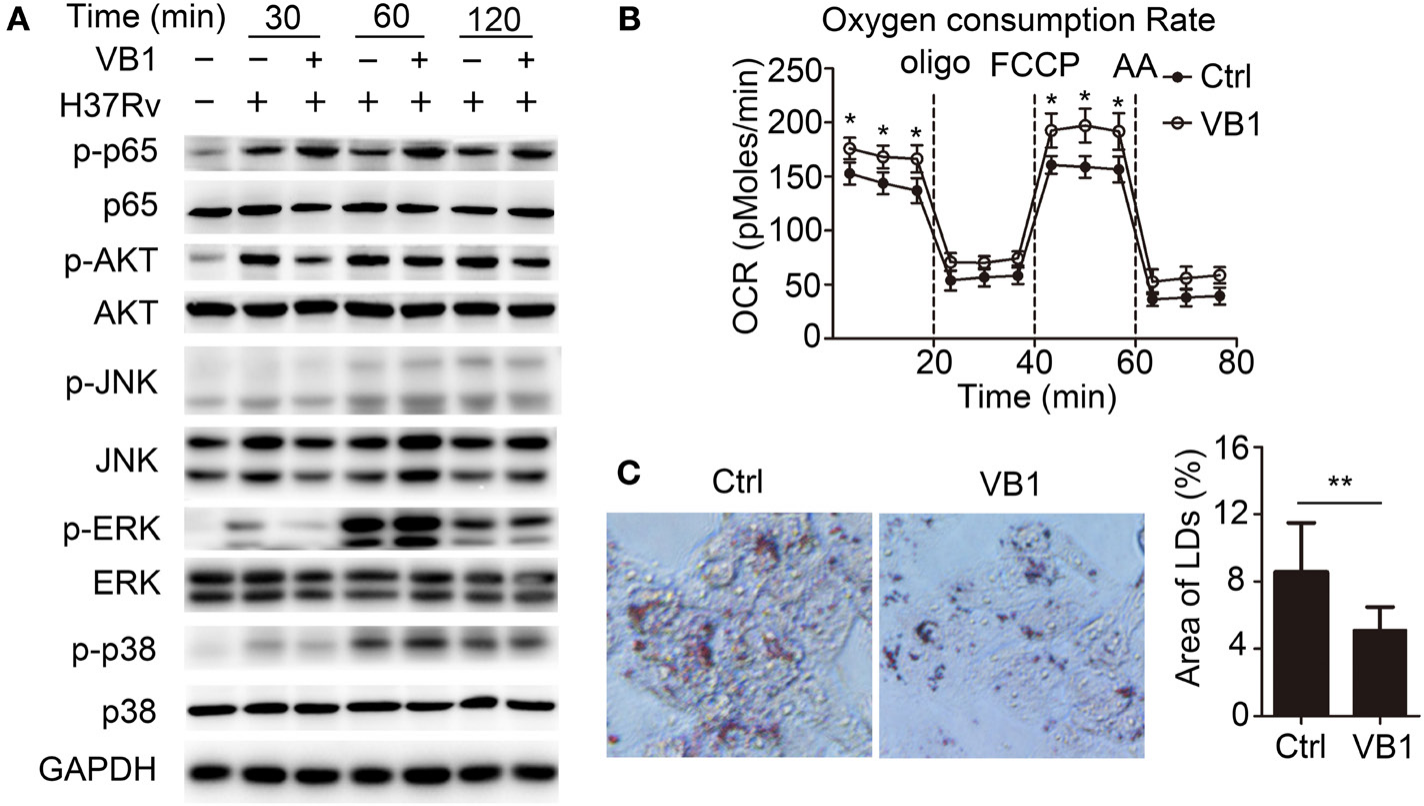
The role of vitamin B1 (VB1) in regulating signaling pathways and mitochondrial respiration. Bone marrow-derived macrophages (BMDMs) were pretreated with VB1 or phosphate buffer saline (Ctrl) for 24 h followed by *Mycobacterium tuberculosis* H37Rv infection (MOI 5). **(A)** Western blot analysis of the phosphorylation status of p65, AKT, JNK, ERK, and p38. GAPDH is as an internal control. These results are from a representative experiment (*n* = 3). **(B)** Oxygen consumption rate (OCR) of BMDMs. O_2_ consumption was normalized to protein content. oligo, oligomycin; FCCP, carbonyl cyanide 4-(trifluoromethoxy) phenylhydrazone; AA, antimycin A. **(C)** BMDMs were stained with Oil Red O to stain the lipid droplets. Staining was assessed by bright field microscopy and Image J software was used to calculate the total area of Oil Red O-stained droplets per cell. Original magnification ×600. Values are mean ± SD of all cells in 20 randomly selected micrographs from each group. *P < 0.05 and **P < 0.01. Data are representative of three independent experiments with similar results.

Polarization and cytokine expression of macrophages are known to be regulated by NF-κB-, PI3K-AKT-, and MAPK- (ERK1/2, p38, and JNK) dependent signaling pathways. We evaluated the role of VB1 in regulating these signaling pathways during mycobacterial infection. We found that phosphorylation of NF-κB p65 was enhanced but phosphorylation of AKT appeared reduced in VB1-treated macrophages (Figure 4A; Figure S3 in Supplementary Material). However, VB1 treatment did not obviously affected ERK1/2, c-Jun N-terminal kinase (JNK), or p38 phosphorylation (Figure 4A; Figure S3 in Supplementary Material). These results demonstrate that VB1 mainly regulates the NF-κB signal in macrophages after mycobacterial infection.

### VB1 Regulated Mitochondrial Respiration and Lipid Metabolism in Macrophages

Vitamin B1 is reported to be indispensable for metabolism in its active form thiamine pyrophosphate. Furthermore, a previous report suggested that metabolic signaling regulates inflammatory signaling. Therefore, we examined whether VB1 regulated NF-κB signal by mediating the metabolism in macrophages. First, we investigated whether VB1 affected the metabolism of macrophages. Indeed, basal and ATP-dependent OCR readings indicated that BMDMs treated with VB1 had significantly higher OCR than untreated group (Figure 4B). In addition, VB1-treated BMDMs contained less lipid bodies than control BMDMs (Figure 4C). Thus, VB1 increases mitochondrial respiration and lipid metabolism in macrophages. Because fatty acids have been shown to modulate the regulation of innate immune response, our data suggest that VB1 may impact macrophage function after MTB infection by regulating lipid metabolism.

### VB1-Mediated Innate Immune Responses Are Mediated Through Modulation of PPAR-γ Signaling

**Figure 5:**
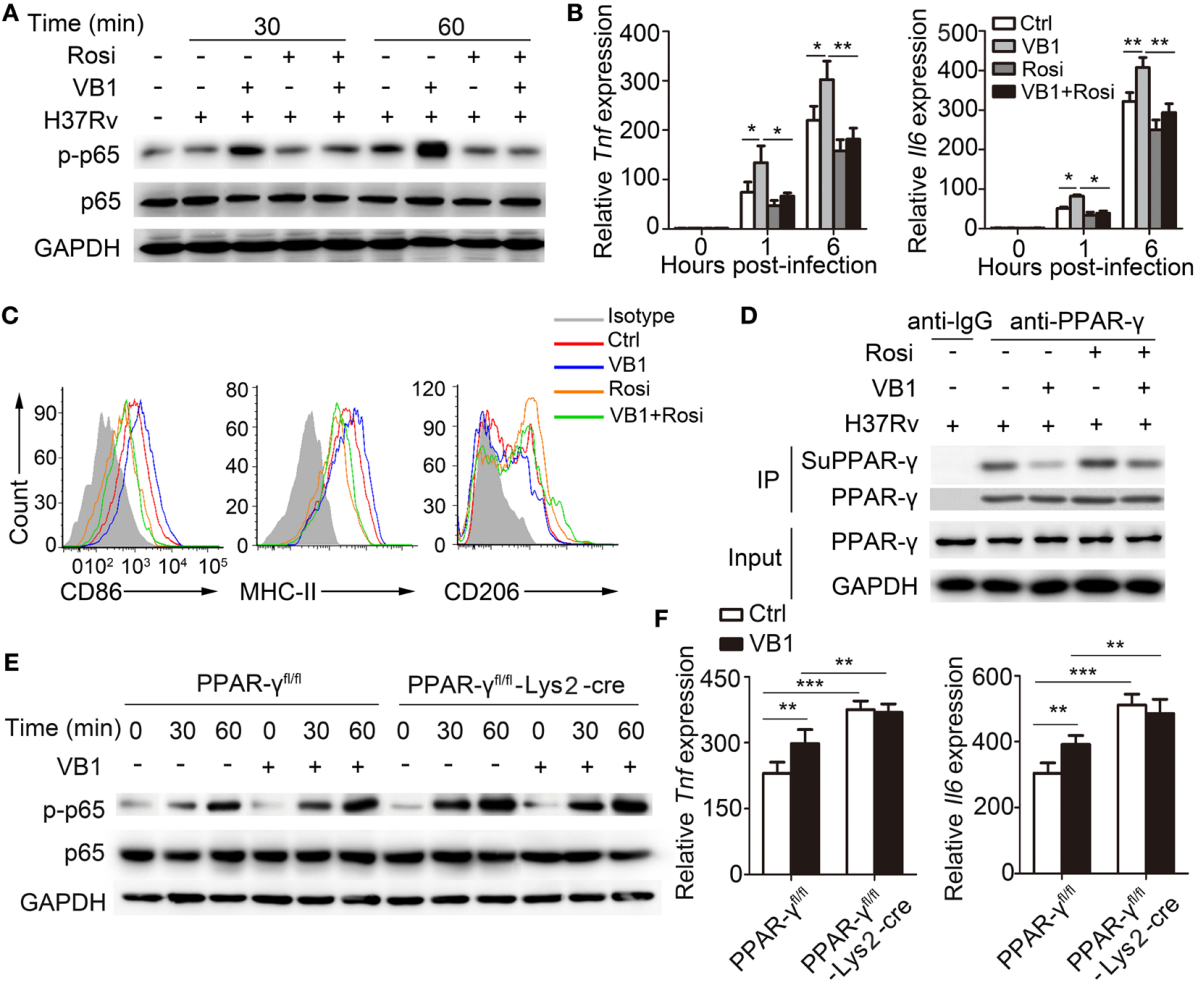
Vitamin B1 (VB1) promoted the innate immune response *via* suppressing SUMOylation of peroxisome proliferator-activated receptor (PPAR-γ). Bone marrow-derived macrophages (BMDMs) were pretreated with phosphate buffer saline, VB1, Rosi, or mixture of VB1 and Rosi for 24 h and then challenged with *Mycobacterium tuberculosis* H37Rv (MOI 5) for indicated time. **(A)** Western blot analysis of the phosphorylation status of p65. GAPDH is as an internal control. **(B)** Tumor necrosis factor α (TNF-α) and interleukin-6 (IL-6) mRNA expression determined by real-time PCR. **(C)** The expressions of CD86, MHC-II, and CD206 were detected *via* flow cytometry after infection at 24 h. **(D)** Immunoprecipitation and immunoblots were performed with indicated antibodies. **(E)** Western blot analysis of the phosphorylation status of p65 in BMDMs from PPAR-γ^fl/fl^ and PPAR-γ^fl/fl^-Lys2-cre mice. GAPDH is as an internal control. **(F)** TNF-α and IL-6 mRNA expression determined by real-time PCR in BMDMs from PPAR-γ^fl/fl^ and PPAR-γ^fl/fl^-Lys2-cre mice. Data shown are the mean ± SD. *P < 0.05, **P < 0.01, and ***P < 0.001. Data are representative of three independent experiments with similar results.

PPARs integrate metabolic and inflammatory signaling in macrophages (19). PPAR-γ, in particular, is known to function as an important “molecular switch” in regulating macrophage immune responses to MTB (20). PPAR-γ, activated by it endogenous ligands, such as polyunsaturated fatty acids or fatty acid derivatives, sequesters the p65 subunit of NF-κB complex and prevents NF-κB-dependent regulation of genes (19). To determine whether VB1 regulation of the NF-κB signal depends on PPAR-γ, we used rosiglitazone, an agonist of PPAR-γ, to activate PPAR-γ after VB1 treatment in macrophages with H37Rv infection. We found that rosiglitazone neutralized the activation of NF-κB p65 induced by VB1 (Figure 5A; Figure S4 in Supplementary Material). The PCR products of IL-6 and TNF-α were no longer increased in VB1-treated BMDMs after rosiglitazone addition (Figure 5B). Furthermore, increased CD86 and MHC-II expression induced by VB1 were abrogated after rosiglitazone treatment, while CD206 expression was recovered after rosiglitazone treatment (Figure 5C; Figure S5A in Supplementary Material). Likewise, the amount of nitrate and activity of arginase-I would be not different between VB1-treated and mix of VB1 and rosiglitazone-treated groups (Figure S5B in Supplementary Material). A previous study showed that ligand-dependent SUMOylation mediates the initial step of transrepression of inflammatory response genes by PPAR-γ (22). Our results showed that the expression of PPAR-γ was not changed after VB1 treatment in BMDMs (Figure 5D—input), but SUMOylation of PPAR-γ was suppressed (Figure 5D—IP; Figure S6A in Supplementary Material).

To assess whether the regulatory function of VB1 in macrophages is dependent on PPAR-γ, we generated and analyzed BMDMS from PPAR-γ^fl/fl^-Lyz2-Cre mice. We found that phosphorylation level of NF-κB p65 was enhanced in BMDMs from PPAR-γ-Lys2-cre mice after MTB H37Rv infection, but it was not different between VB1-treated and control groups (Figure 5E; Figure S6B in Supplementary Material). Similarly, TNF-α and IL-6 mRNA levels were increased in PPAR-γ-deficient BMDMs, but they were not further increased with VB1 treatment (Figure 5F). These results suggest that VB1-mediated innate immune responses are dependent on PPAR-γ.

### VB1 Limits Mycobacterial Growth in Macrophages

**Figure 6:**
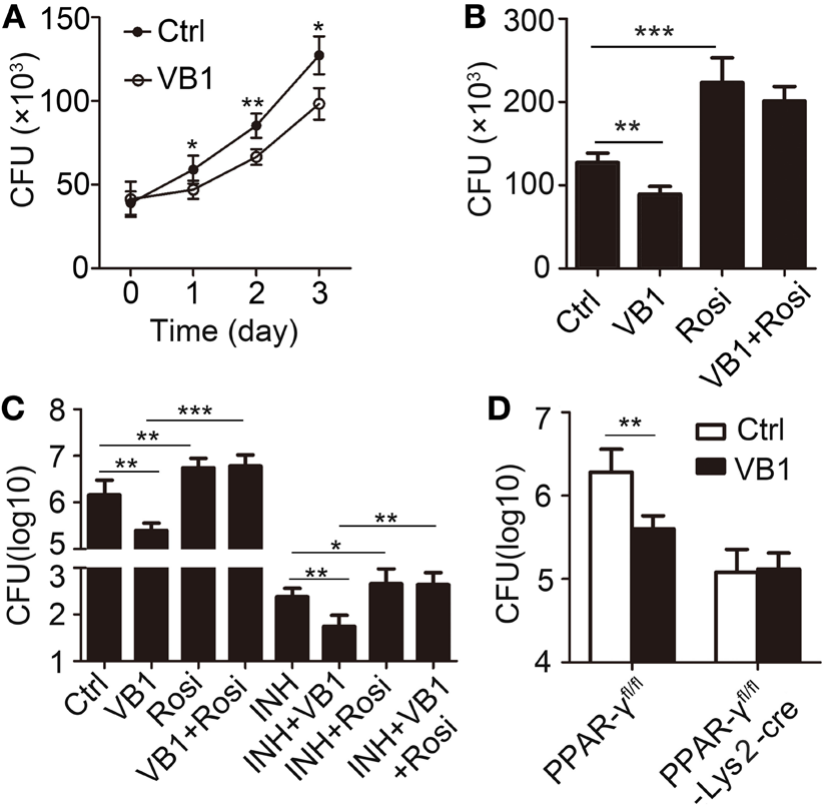
The anti-bacillus effect of vitamin B1 (VB1) in macrophages. **(A,B)** Bone marrow-derived macrophages were pretreated with phosphate buffer saline, VB1, Rosi, or mixture of VB1 and Rosi for 24 h and then challenged with *Mycobacterium tuberculosis* H37Rv (MOI 5) for 1 h. **(A)** Intracellular viable bacteria were detected with colony-forming unit (CFU) assays at 0, 1, 2, 3 day post-infection. **(B)** Intracellular viable bacteria were detected with CFU assays at 72 h post-infection. **(C,D)** PPAR-γ^fl/fl^ or PPAR-γ^fl/fl^-Lys2-cre mice were infected with H37Rv (~200 bacteria/mouse). Oral administration with water (Ctrl), VB1, INH, or Rosi (*n* = 5 mice/group) was started from the day after infection (day 1) and continued for 2 weeks. CFUs were obtained from the lung cell lysates by serial dilution and plating on 7H10 agars in triplicate. The colonies were counted after 4 weeks. **(C)** C57BL/6J mice. **(D)** PPAR-γ^fl/fl^ or PPAR-γ^fl/fl^-Lys2-cre mice. Data shown are the mean ± SD. *P < 0.05, **P < 0.01, and ***P < 0.001. Data are representative of three independent experiments with similar results.

The data we have shown thus far indicated that VB1 regulated functions of macrophages in a PPAR-γ-dependent manner. We further examined the role of VB1 in inhibiting MTB H37Rv growth in macrophages. Treatment of BMDMs with VB1 during MTB H37Rv infection reduced mycobacterial CFU inside the cells 1, 2, and 3 days post-infection (Figure 6A). The number of intracellular viable bacilli in mock-treated BMDMs was approximately 1.5-fold greater than in VB1-treated cells on 3 days post-infection (Figure 6A). However, BMDM survival was not obviously affected by VB1 treatment (Figure S7A in Supplementary Material). Such negative effects of VB1 on MTB growth/survival was diminished when BMDMs were simultaneously treated with rosiglitazone (Figure 6B). Moreover, BMDMs pretreated with VB1 showed similar abilities of phagocytosis of fluorescently labeled MTB H37Rv, suggesting that VB1 had no major effects on cell association of MTB H37Rv with macrophages (Figure S7B in Supplementary Material).

To investigate if VB1 exerted its protective role in a PPAR-γ-dependent manner, we examined MTB H37Rv burden in the lung after oral administration of VB1 with or without rosiglitazone for 2 weeks. We found that rosiglitazone treatment increased MTB burden in the lung and diminished the protective effect of VB1 on MTB *in vivo* (Figure 6C). Furthermore, mice with PPAR-γ-deficiency in myeloid cells showed stronger antibacterial ability than WT control mice, but VB1 did not additively enhance protective immune responses in PPAR-γ-deficiency mice against MTB (Figure 6D). Collectively, these results indicate that VB1 is instrumental in limiting growth of intracellular mycobacteria in macrophages and *in vivo* that is dependent on proper PPAR-γ activity.

## Discussion

Since the development of anti-tuberculosis drugs, adjunct tuberculosis therapies, including therapeutic vaccines, vitamin supplementation, and/or repurposing of drugs targeting biologically and clinically relevant molecular pathways, have received considerable attention (13, 15). Previous studies showed that thiamine pyrophosphate, a VB1 derivative, protects retinal tissues from ethambutol-induced oxidative damage (17), that VB1 inhibits the production of cytokines and increases the anti-inflammatory activity of a corticosteroid in a chronic model of inflammation induced by complete Freund’s adjuvant (23), and that VB1 reduced serum pro-inflammatory cytokines in adjuvant-induced arthritis and DEN-induced hepatic cancer (24, 25). However, no study examining the immune regulatory mechanism of VB1 in MTB infection has been reported. We observed increased TNF-α in lung homogenate of VB1-treated mice with MTB infection, which did not agree with the results of previous studies. We also found that VB1 supplementation promoted NF-κB signaling and IL-6 and TNF-α production in macrophages. Thus, the function of VB1 in different cell types may differ. Further studies are required to clarify this possibility.

A hallmark of MTB infection is the differentiation of infected macrophages into lipid-rich foam cells (26). These cells accumulate lipid droplets, which are lipid storage organelles required for intracellular bacillary growth (27). A previous study showed that VD treatment abrogates infection-induced accumulation of lipid droplets of infected macrophages and is beneficial for suppressing the growth of MTB in macrophages (28). Thiamine pyrophosphate, the active form of VB1, is a cofactor present in all living systems and is indispensable for metabolism (29). Our results showed that VB1 supplementation in macrophages increased mitochondrial respiration and lipid metabolism. Thus, it is possible that VB1 exerts its protective roles during MTB infection by affecting macrophage lipid metabolism.

Peroxisome proliferator-activated receptor-γ integrates metabolic and inflammatory pathways (19) and functions as an important “molecular switch” in regulating macrophage immune responses to MTB (20, 30). In this study, we found that VB1 regulated the NF-κB signal in a PPAR-γ-dependent manner. VB1 supplementation suppressed the activation of PPAR-γ, whereas an agonist of PPAR-γ neutralized the antimycobacterial effect of VB1. This is consistent with the results of a previous study showing that activation of PPAR-γ decreased TNF production and promoted the intracellular growth of MTB (31, 32). In addition, we found that VB1 prompted the polarization of macrophages into M1 macrophages (classically activated macrophages) *in vitro* and *in vivo*, which is beneficial for suppressing mycobacteria growth (33). A previous study showed that PPAR-γ has a vital role in the polarization of macrophages with MTB infection (31, 34). In agreement with their study, we found that the agonist of PPAR-γ reduced the increased CD86 and MHC-II expression but promoted CD206 expression.

Taken together, these findings indicate that VB1 can significantly inhibit MTB growth *in vitro* and *in vivo* by regulating innate immunity. VB1 may exert its immune regulation function during MTB infection *via* multiple mechanisms that include modulating NFκB signaling and lipid metabolism. We suggest that, in clinical trials involved with VB1 supplement, the immune regulation effects of VB1 should be monitored.

## Ethics Statement

All animal experiments in this study were carried out in accordance with the recommendations in the Guide for the Care and Use of Laboratory Animals of the National Institutes of Health. All experimental protocols were reviewed and approved by the Medical Ethics Board and the Biosafety Management Committee of Southern Medical University (approval number L2015123).

## Author Contributions

SH and LM designed research; SH, WH, XD, YF, YY, CH, SL, QSW, and YH conducted research; SH, WH, XZ, CZ, QW, and LM analyzed data; SH, X-PZ, and LM wrote the paper. LM had primary responsibility for final content. All authors read and approved the final manuscript.

## Conflict of Interest Statement

The authors declare that the research was conducted in the absence of any commercial or financial relationships that could be construed as a potential conflict of interest.
