# Supplementary material for: Vitamin B1 Helps to Limit Mycobacterium tuberculosis Growth via Regulating Innate Immunity in a Peroxisome Proliferator-Activated Receptor-γ-Dependent Manner
Source: Front Immunol. 2018 Aug 16;9:1778. doi: 10.3389/fimmu.2018.01778 (PMC6106772; doi:10.3389/fimmu.2018.01778)
Supplement: Supplementary file 1 [file Data_Sheet_1.docx]

**Supplememtary Material**

**Vitamin B1 Helps to Limit *Mycobacterium tuberculosis* Growth via Regulating Innate Immunity in a PPAR-γ Dependent Manner**

Shengfeng Hu^1, #^, Wenting He^1, #^, Xialin Du^1^, Yulan Huang^1^, Yuling Fu^1^, Yalong Yang^1^, Chuxuan Hu^1^, Silin Li^1^, Qinshu Wang^1^, Qian Wen^1^, Xinying Zhou^1^, Chaoying Zhou^1^, Xiao-Ping Zhong^1,2^, Li Ma^1,^*

^1^Institute of Molecular Immunology, School of Laboratory Medicine and Biotechnology, Southern Medical University, Guangzhou 510515, China.

^2^Department of Pediatrics, Division of Allergy and Immunology, Duke University Medical Center, Durham, NC 27710, USA.

# These authors contributed equally to this work.

*** Corresponding Author:**

Li Ma, M.D., Ph.D., Institute of Molecular Immunology, School of Laboratory Medicine and Biotechnology, Southern Medical University, Guangzhou 510515, China.

E-mail: [maryhmz@126.com](mailto:maryhmz@126.com). Telephone number: 86 20 61648322. Fax number: 86 20 61648322.

**Supplementary Figures and Figure Legends**

**Supplement figure 1**

**
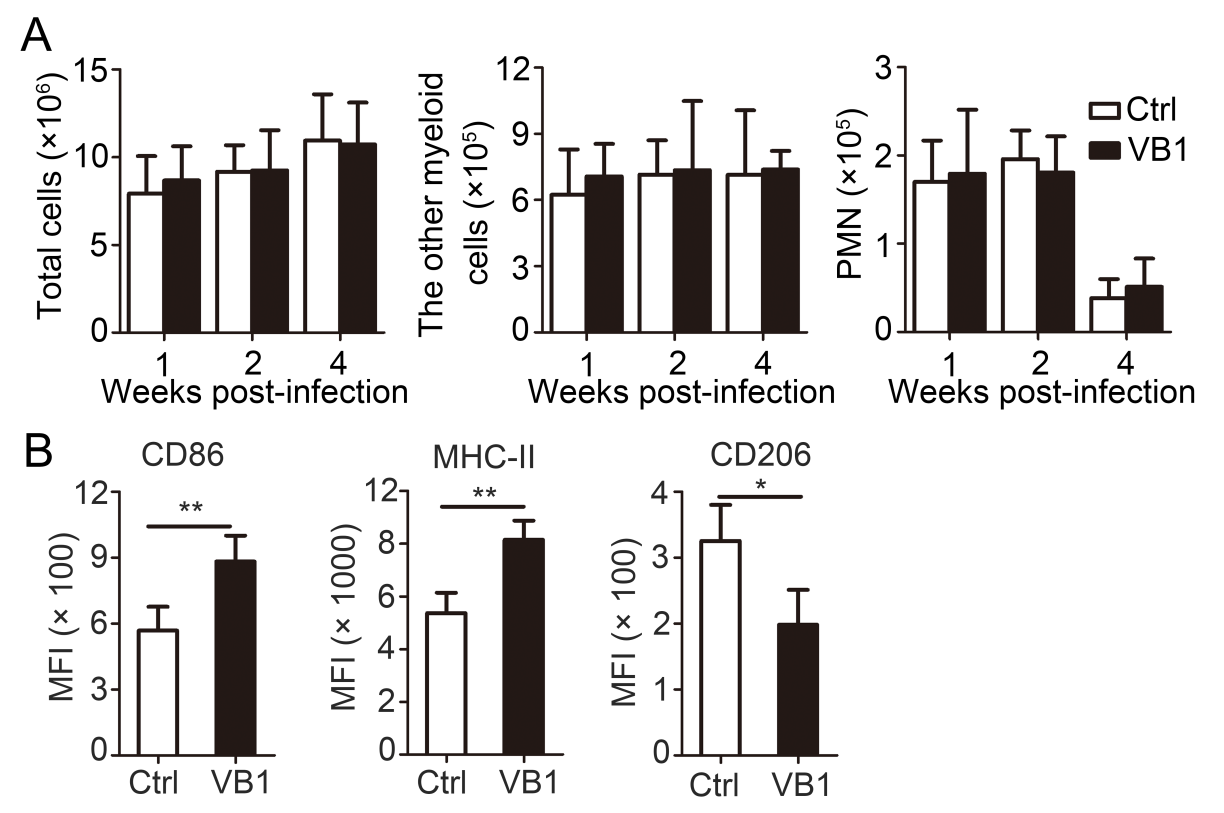
**

**Figure S1 The anti-bacillus effect of VB1 in mice with MTB infection.** Lung cells from H37Rv-infected mice treated with VB1 or untreated were harvested at 1 week, 2 weeks and 4 weeks after infection. (A) The numbers of total, the other myeloid cells and polymorphonuclear cells (PMNs) in lungs were shown. (B) Lung cells from H37Rv-infected mice treated with VB1 or untreated were harvested 4 weeks after infection. The expressions of CD86, MHC-II and CD206 on macrophages were assessed as mean fluorescence intensity (MFI). Data shown are the mean ±SD. **P* < 0.05 and ***P* < 0.01. Data are representative of three independent experiments with similar results.

**Supplement figure 2**

**
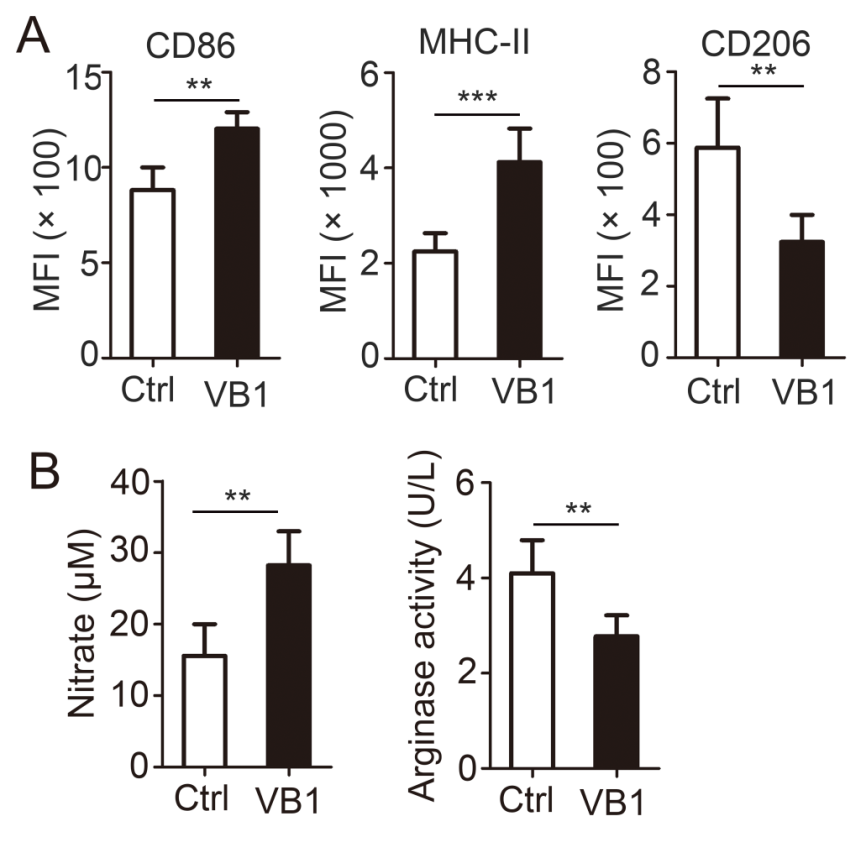
**

**Figure S2 The proinflammatory effect of VB1 on BMDMs after MTB infection** BMDMs were pretreated with phosphate buffer saline and VB1 for 24 hour and then challenged with MTB H37Rv (MOI 5). (A) The expressions of CD86, MHC-II and CD206 were detected via flow cytometry after infection at 24 hours. The expressions of CD86, MHC-II and CD206 were assessed as mean fluorescence intensity (MFI). (B) Concentrations of nitrate in the supernatant cultured and arginase activity in BMDMs for 24 h. Data shown are the mean ± SD. ***P* < 0.01 and ****P* < 0.001. Data are representative of three independent experiments with similar results.

**Supplement figure 3**


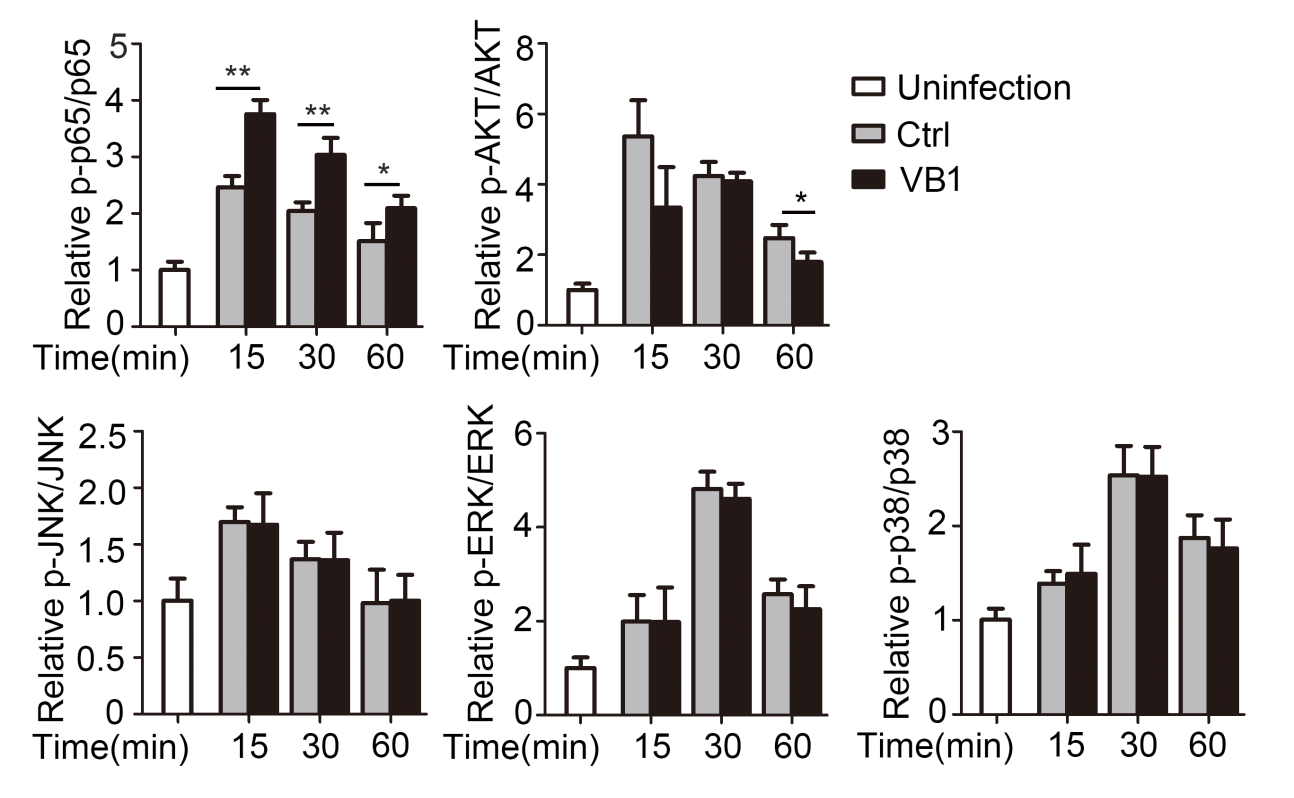


**Figure S3 Densitometry quantification of band intensity of Figure 2**. BMDMs were pretreated with VB1 followed by MTB H37Rv infection for appointed time. Western blot analysis of the phosphorylation status of NF-κB, AKT, JNK, ERK and p38. Data shown are the mean ±SD. **P* < 0.05 and ***P* < 0.01. Data are representative of three independent experiments with similar results.

**Supplement figure 4**

**
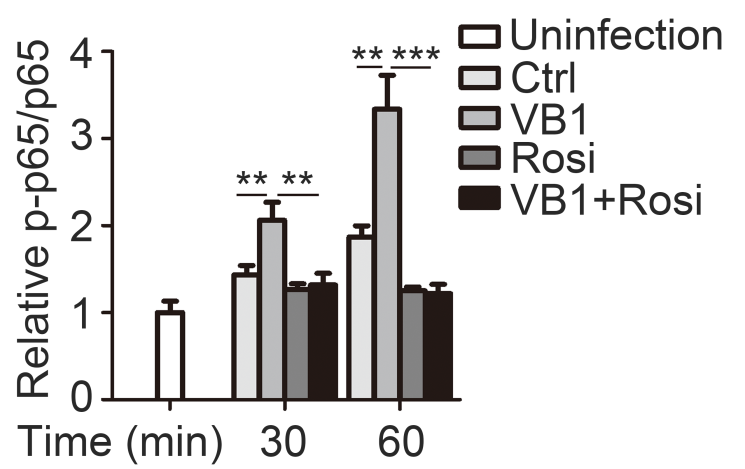
**

**Figure S4 Densitometry quantification of band intensity of Figure 5A**. Densitometry quantification of band intensity of Figure 5A.Data shown are the mean ±SD. ***P* < 0.01 and ****P* < 0.001. Data are representative of three independent experiments with similar results.

**Supplement figure 5**

**
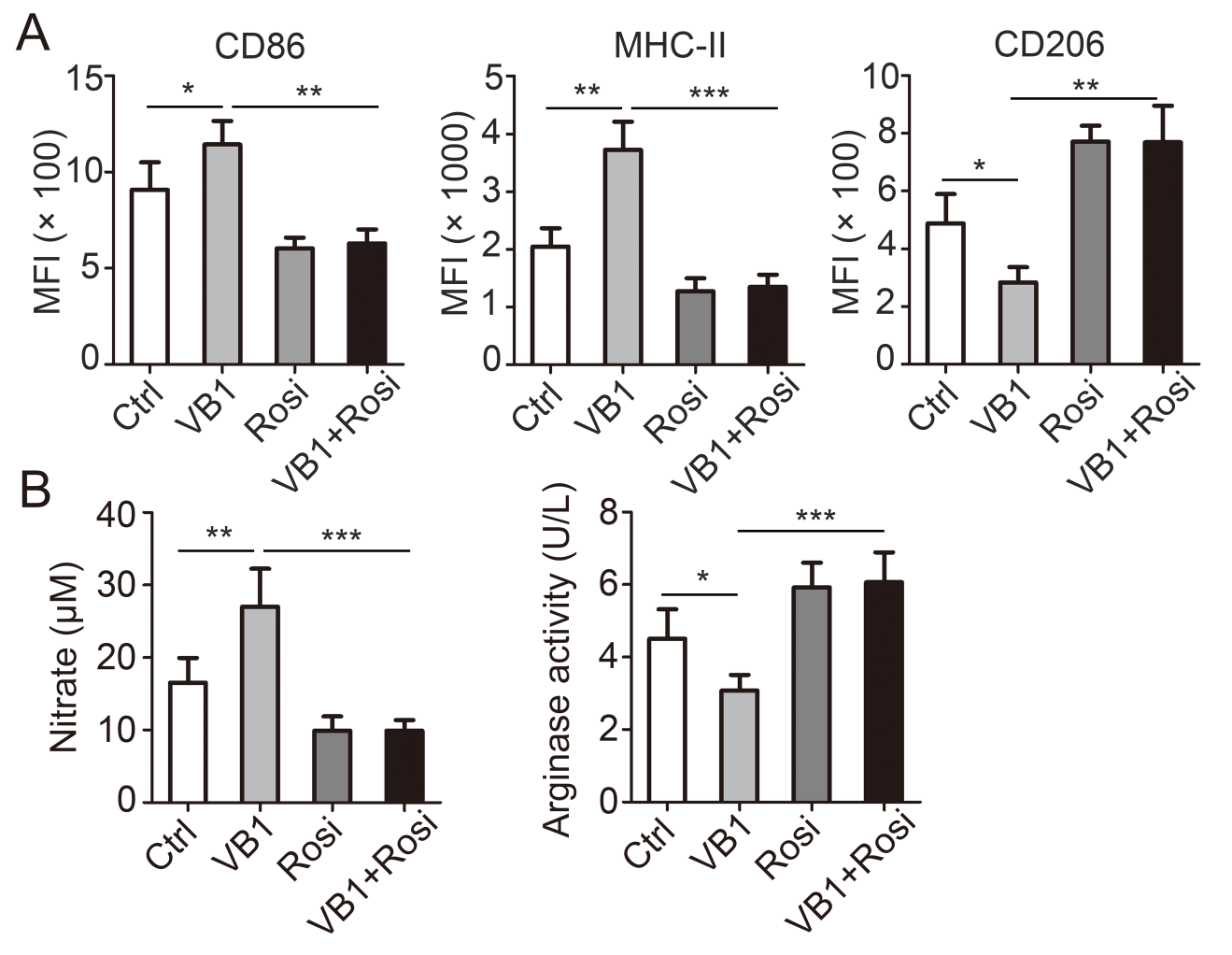
Figure S5 VB1 promoted the innate immune response via suppressing SUMOylation of PPAR-γ.** BMDMs were pretreated with phosphate buffer saline, VB1, Rosi or mixture of VB1 and Rosi for 24 hour and then challenged with MTB H37Rv (MOI 5). (A) The expressions of CD86, MHC-II and CD206 were detected via flow cytometry after infection at 24 hours. The expressions of CD86, MHC-II and CD206 were assessed as MFI. (B) Concentrations of nitrate in the supernatant cultured and arginase activity in BMDMs for 24 h. Data shown are the mean ± SD. **P* < 0.05, ***P* < 0.01 and ****P* < 0.001. Data are representative of three independent experiments with similar results.

**Supplement figure 6**


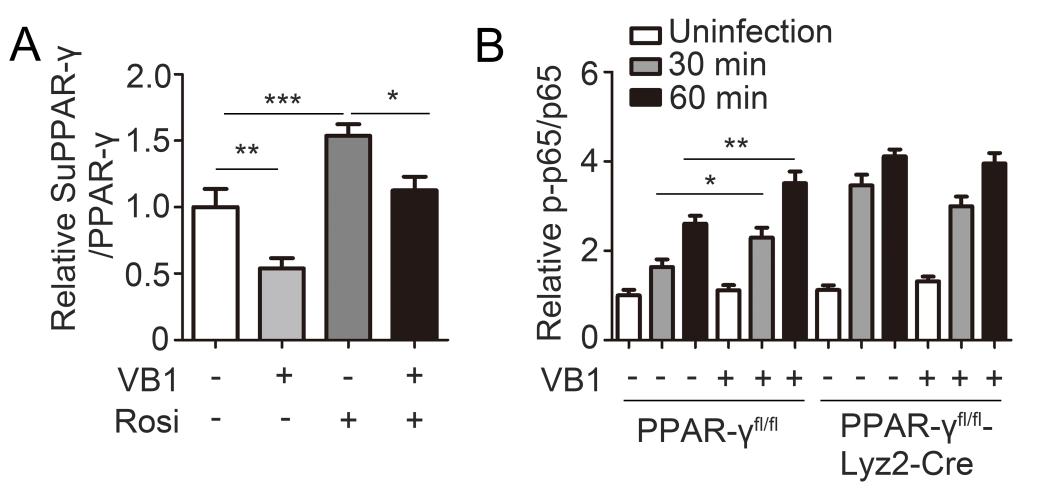


**Figure S6 Densitometry quantification of band intensity of Figure 5**. **(A)** Densitometry quantification of band intensity of Figure 5D. **(B)** Densitometry quantification of band intensity of Figure 5E. Data shown are the mean ±SD. **P* < 0.05, ***P* < 0.01 and ****P* < 0.001. Data are representative of three independent experiments with similar results.

**Supplement figure 7**

**
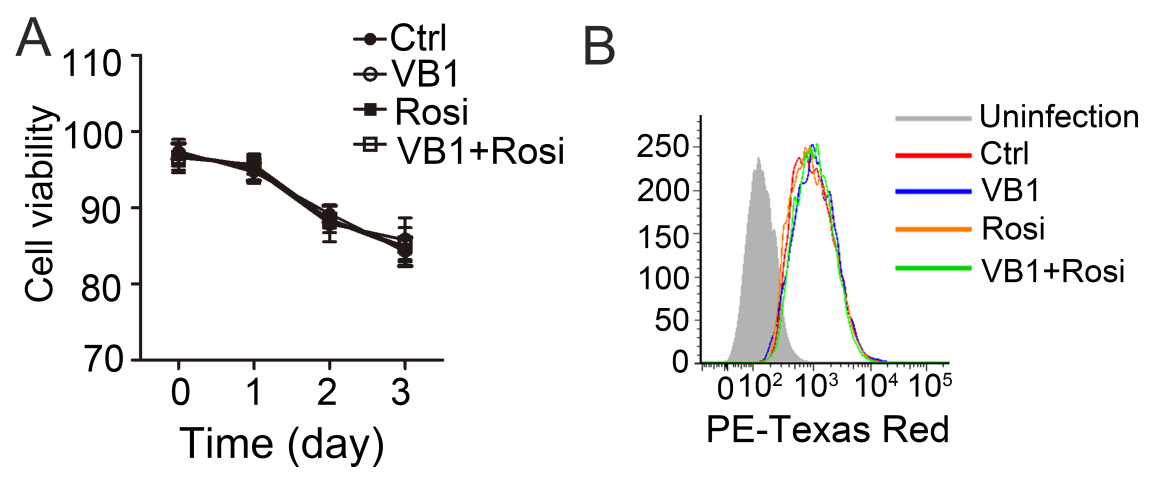
**

**Figure S7 Effects of VB1 on cell viability and phagocytosis of mycobacteria after mycobacterial infection.** **(A)** BMDMs were pretreated with phosphate buffer saline, VB1, Rosi or mixture of VB1 and Rosi for 24 hour and then challenged with MTB H37Rv (MOI=5) for 1 hour. The infected cells were washed extensively with phosphate buffer saline to remove extracellular mycobacteria. Cells were collected after incubated for the indicated time and stained with propidium iodide (PI) to test cell viability. The percentage of cells viability was shown. **(B)** BMDMs were pretreated with phosphate buffer saline, VB1, rosiglitazone (Rosi) or mixture of VB1 and Rosi for 24 hour and then challenged with Texas-Red-labeled MTB H37Rv (MOI 5) for 1 hour. Phagocytosis of MTB H37Rv was determined by flow cytometry. Data shown are the mean ±SD. Data are representative of three independent experiments with similar results.

**Supplement Materials and Methods**

**Staining with Propidium iodide (PI)**

BMDMs were infected with MTB H37Rv at an MOI of 5. After 1hr incubation at 37 °C, the infected cells were washed extensively with PBS to remove extracellular mycobacteria, and the infected cells were incubated for indicated time. Cells were collected and stained with PI (Sangon Biotech, E607306) according to the manufacturer’s instructions, and measured by flow cytometry.
